# A Qualitative Description of Community Participation in Water and Sanitation Activities in the Control of Schistosomiasis in Nyalenda B, an Informal Settlement in Kisumu City, Western Kenya

**DOI:** 10.24248/EAHRJ-D-18-00032

**Published:** 2019-07-30

**Authors:** Rosemary M Musuva, Gladys O Odhiambo, Vincent O Atuncha, Elizabeth T Mutete, Maurice R Odiere, Bernard Abong'o, Jane Alaii, Pauline NM Mwinzi

**Affiliations:** a Neglected Tropical Diseases Branch, Centre for Global Health Research, Kenya Medical Research Institute, Kisumu, Kenya; b Public Health Department, School of Public Health and Community Development, Maseno University, Maseno, Kenya; c Context FACTOR Solutions, Nairobi Kenya

## Abstract

**Background:**

Community participation is central to the success of primary health care. However, over 30 years since the Alma Ata declaration, the absence of universal community participation remains a major obstacle to combating all types of diseases. This study investigated community participation in water and sanitation activities towards schistosomiasis control in Nyalenda B, an informal settlement in Kisumu City.

**Methods:**

Eight key informant interviews (KIIs) and 8 focus group discussions (FGDs) were conducted. Additionally, data on NGOs dealing with water and sanitation activities in Kisumu was collected from the local NGO registration Board. Qualitative data was organised into themes and concepts and analyzed using Atlas.ti.

**Results:**

Most participants felt that project implementers did not involve them in key levels of project implementation, leading to unsustainable projects and unacceptance from the community. Community structures identified that could be used as avenues of engaging the community in improving water and sanitation situation included the use of organised groups, such as youth, gender-based, farmers and HIV support groups, and merry-go-rounds. Factors mentioned that hindered community participation included negative attitude from community members, poor monitoring and evaluation strategies, limited disclosure of project details, and overdependence from the community.

**Conclusion:**

Effective community participation in water and sanitation activities requires a multipronged paradigm that incorporates a change of attitude from the community, information sharing and consultation, improved monitoring and evaluation, transparency and accountability.

## INTRODUCTION

Large populations in the developing countries do not have ready access to adequate water and sanitation even though it is universally accepted as a basic need. This situation is worse in informal urban settlements in developing countries where environmental contamination, often associated with improper waste and excreta management, is widespread.^1^ The spread of many infectious diseases, including cholera, typhoid, hepatitis, polio, cryptosporidiosis, soil-transmitted helminths (STH) and schistosomiasis (Bilharzia), is associated with faecal matter and poor hygiene. About one-third of deaths in developing countries are caused by the consumption of contaminated water and, on average, as much as one-tenth of each person's productive time is sacrificed to water-related diseases.^2^ In addition, 10% of the population in developing countries is severely infected with intestinal worms related to poor faecal waste management.^3^ Worm infestations continue to be a major public health and socio-economic concern despite efforts and improvements that have been made to reduce helminth transmission worldwide.^4^ An estimated 207 million people in 74 countries are infected with bilharzia (schistosomiasis),^5^ 90% of whom reside in sub-Saharan Africa.^6^

In Kenya, over 9.1 million people are infected with schistosomiasis^7^ despite the availability of an effective and safe drug. While schistosomiasis has generally always been considered a rural phenomenon, there is increasing evidence on the existence and active transmission of the disease in urban settings.^8-10^ This is because of high populations in urban settings which facilitate high rates of disease transmission, larval development sites and poor waste disposal mechanisms.^11^ This creates a critical need to scale up control interventions against schistosomiasis in urban settings. Schistosomiasis can be controlled using 3 key approaches: improved sanitation, health education and treatment using available and safe drugs. Other control measures include targeting vector snails and avoiding contact with infected waters.^12^ Regular administration of anthelmintic drugs has strongly been advocated for by the World Health Organization (WHO) as an effective control strategy. The role of community participation in water and sanitation activities in the control of schistosomiasis has been advocated for, though few studies have looked at this in detail.

Although studies prove that improving access to clean water, changing hygiene, behaviour and proper waste disposal go a long way in preventing parasitic diseases, very little emphasis has been placed on this.^13-15^ Community participation not only enhances control efforts but also guarantees their sustainment. In community participation, rather than be mere recipients, beneficiaries are actively involved in the planning and execution of the development projects. They also have a say in the nature and direction of the projects.^16^ A good example is community-directed mass treatment, which has successfully been used for the control of diseases such as lymphatic filariasis and onchocerciasis and is currently being tried for schistosomiasis.^17,18^ The strategy is well-accepted by communities since local members are fully involved and take responsibility for delivery of treatment.^19^ Schistosomiasis control efforts advocated for by the WHO largely focus on chemotherapy. However, these efforts can be augmented by involving communities in control interventions targeting water, sanitation and chemotherapy. Since community structures vary in different settings, they can influence the outcome of control efforts against schistosomiasis. This study therefore sought (1) to describe awareness on schistosomiasis and other water and sanitation-related diseases among community members; (2) to assess the water and sanitation situation in the area; and (3) to identify structures and issues that affect community participation in water and sanitation activities in the control of schistosomiasis in Nyalenda B. Understanding community participation in water and sanitation activities in the control of schistosomiasis in informal settlement areas is critical in designing, implementing and ensuring the sustainment of the control interventions.

## METHODS

### Conceptual Framework

The study adopted Laodicah's model for community participation^20^ that argues that for communities to be fully involved, there are 4 stages that project implementers need to bear in mind. First, information sharing stage is a one-way information sharing process from project managers to community members. This is done in the case of sharing preliminary findings. Second, the consultation stage involves programme managers not only sharing information with community members but also seeking their opinion on proposed projects. Third, the decision making stage involves engaging the community on project design, implementation and objectives. Fourth, the initiating-action stage is a proactive process where community members take charge of the health gaps in their community and are more involved in the implementation stage of projects.

### Study Area

The study was conducted in Nyalenda B, an informal settlement in Kisumu City, situated along the shores of Lake Victoria in western Kenya. Nyalenda B is 1 of the 8 informal settlements in Kisumu City, with an area of 6.1 km^2^ and an estimated population of 32,430, population density of 5,317 people/km^2^.^21^ It comprises 8,561 households with a total population: 32,430 people.^20^ Nyalenda B is divided into 5 health units, which are further subdivided into 9 subunits that are either headed by 1 or 2 village elders depending on the size of the subunit.

Kisumu, like the majority of fast-growing cities, faces various challenges such as overcrowding as well as water and sanitation issues that are common with urbanisation. For example, it faces an acute water shortage, and only 40% of the population has access to piped water. The majority of residents rely on unprotected wells and the lake which are subject to high degrees of contamination due to the rampant use of pit latrines and high water tables.^17^ Schistosomiasis being a waterborne infection, It is, therefore, no surprise that the presence of intermediate host snails for schistosomiasis have been reported from some of the frequently accessed water points.^10^ Other studies in the area have also found high prevalences of schistosomiasis^9^ A map of the study area is shown below in the Figure.

**FIGURE:**
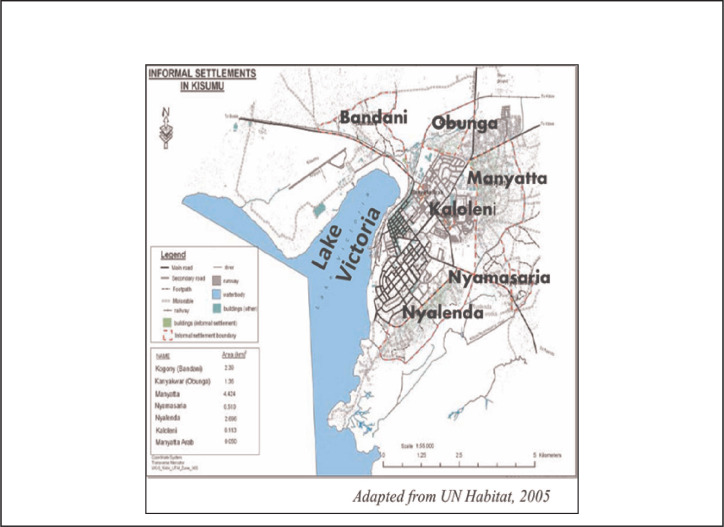
Map of the Study Area

### Study Design

This was a cross-sectional qualitative study involving 8 FGDs with local community members and 8 KIIs with individuals in various professions or assuming leadership roles in the community. Purposive sampling was used to select the respondents for the KIIs. The interviews were conducted in the participants' offices or rooms within their working environment. Each FGD with a maximum of twelve participants each were conducted. These were categorised by gender and age. The FGDs were conducted in classrooms in primary schools located within Nyalenda B. Participants were screened at the venue before commencement of the discussion to ensure eligibility and avoid selection bias. Participants for FGDs had to be adults aged 18 years and over, residents of Nyalenda B, and must have lived there for at least 6 months. A semi-structured FGD guide developed by the research team and pilot tested in Obunga, an informal settlement which experiences similar water safety and sanitation issues as Nyalenda B, was used. This guide covered: knowledge on signs and symptoms, community structures, issues and challenges in community participation and the roles of gender and disability. Both English and Swahili were used for data collection.

### Data Analysis

Voice data from both the KIIs and FGDs were tape-recorded and later transcribed. Transcripts that were in Swahili were translated and back-translated to English to ensure that both versions carried the same meanings. Data coding was conducted by the research team. The coding structure evolved inductively with the codes from the narrative data of earlier interviews informing subsequent coding of the following interviews supplemented with field notes from the interviewer and note-taker. A coding frame was developed through open coding, a word-by-word analysis used to identify, name, and categorise explanations and descriptions of the day-to-day reality of participants as related to their perspectives on the water and sanitation situation in their community. Consensus on the coding frame was obtained through discussions between the 2 research assistants who had also participated in data collection. Each transcript was then examined to identify texts relevant to the coding frame. Quotes were later retrieved from the output monitor and arranged according to themes. Our data was validated by triangulation – verification of transcripts with the audio files, and discussions on the coding systems until agreements were reached.

### Ethical Considerations

The study was part of a larger project evaluating the effectiveness of Community Directed Intervention (CDI) strategy in increasing access to treatment for schistosomiasis and soil-transmitted helminths in Nyalenda. This larger study was reviewed and approved by the Scientific Ethical Review Committees (ERC) of the Kenya Medical Research Institute (KEMRI, SSC # 1841) and from the ethics board of Maseno University. Additional clearance for our study was obtained from the Provincial administration and Ministry of Public Health and Sanitation (MOPHS) and the Municipal Council of Kisumu.

The purpose of the study and its objectives were explained to local authorities, opinion leaders, village elders and community members. Consent was obtained from respondents. Participants were assured of the confidentiality of information during data collection. All personal identifiers were excluded during the discussion. FGD participants were given assigned numbers to anonymise the voice data further.

## RESULTS

Eight KIIs were carried out. Additionally, 8 FGDs were conducted, with a total number of 85 participants. Of these, 44 were females, and 41 were males. A few (7%) of the individuals had only elementary education – all of these participants were between 29 and 43 years of age. Nearly half (47%) of the participants had attended high school, while 42% reported having some postsecondary education. The majority of adult men were businessmen and fishermen, while most of the women were fishmongers. Table 1 and Table 2 show the respondents' characteristics.

**TABLE 1.** Characteristics of Key Informant Interview Participants

Age, years	Gender	Education level	Representation
59	Male	Postsecondary	Nurse
38	Male	Secondary	Beach management
54	Female	Secondary	Farmer
52	Female	Secondary	Group leader
45	Female	Postsecondary	Community health worker
30	Female	Primary	Group leader
52	Male	Primary	Village elder
44	Female	Secondary	Church leader

**TABLE 2.** Characteristics of Focus Group Discussion Participants

		Age, years	
Participant Category	n	Mean	Range
**Male Adult**	20	38.1	22-60
**Female adult**	20	35	22-47
**Male youth**	21	22.5	18-29
**Female youth**	24	21	18-29
**Total**	85		
**Education level**			
Primary	9		
Secondary	40		
Post secondary	36		
**Group representation**			
Volunteer	28		
Group leader	28		
Community health worker	21		
Teacher	3		
Church Leader	2		
Unknown	3		

### Awareness About Water and Sanitation-Related Diseases and Infections

When the participants were asked about the most common diseases in the area in relation to water and sanitation, malaria, cholera, amoeba and diarrhoea received the most mention with schistosomiasis being brought up only 4 times. This further confirms the neglected aspect of this disease.

A 31-year-old male CBO leader said:

**We have cholera due to poor drainage systems… when it rains, there is stagnant water. You find that these young kids – they play on this stagnant water, and this affects them so much. You find that sometimes they drink this dirty water.**

A 44-year-old woman, also a CBO leader said:

**I think in our community, we are experiencing some waterborne diseases like typhoid. Typhoid is rampant in this community, again at times we experience diarrhoea, this is cholera break out, and these are some common illness. Diarrhoea at times is not all that about water, but it is about sanitation.**

Participants understood the transmission of cholera and its relationship with sanitation but did not mention schistosomiasis. A 32-year-old salaried worker stated:

**You find children they don't go to the pit latrines for fear that they may drop inside the latrine. So, they just drop their faeces outside in the open – the same place you find the mothers also selling vegetables, the flies go from the faeces to the food they are selling, and they also take the food… this is where cholera comes in.**

### Water and Sanitation Situation in Nyalenda B

It was the general view of participants that the water and sanitation situation in Nyalenda B is wanting. Factors mentioned included poor drainage systems, low latrine coverage, broken pipes and leakage of the sewerage system. Capturing the poor drainage systems in the community, a 37-year-old small business owner said:

**We don't have any drainage system in the whole of Nyalenda. What, maybe, the average income earners use is septic tanks and most of these people also don't empty the septic tanks when they are supposed to. Many of them… wait around rainy season; when it is raining, they drain them and the waters from up sweep the waters down the rivers.**

With regards to low latrine coverage, a 44-year-old male farmer said:

**I think even another problem is that the latrine coverage is very low…so they end up defecating on the open places, and you find that when the rain falls, it carries it to the river.**

On broken pipes and leakage of the sewerage system, an adult male said:

**I have ever used water, and later realised it had passed near some toilet, and the pipe had burst in the toilet, and it had passed very deep inside. There is no proper supervision. They are not put up to the standard that is required.**

Leakage of the sewerage system is further captured by the sentiments of a 44-year-old leader of a women's group:

**In this area, some people have dug pit latrines, and sometimes wells are dug near the latrines and water is water; the water in the latrine can flow into the well. When you draw, you may think you are getting clean water from the well, but you will be affected by this.**

This is despite the NGO registration board reporting that there are 43 NGOs in Kisumu dealing with water and sanitation. Additionally, an interview with the assistant chief in Nyalenda B reveals 4 NGOs working on the same. She was quick to add that there are many who continue to work in the area but do not pass by her office to inform her and she only stumbles on this information occasionally, while interacting with villagers or village heads in the subunits.

### Community Participation Process

Participants reported that they were fairly engaged by programme implementers. However, they mentioned that key steps in community participation: Information sharing, consultation and decision making had been left out in most programmes. This, in turn, led to unsustainable projects and unacceptance from the community. On information sharing, a 23-year-old male youth volunteering in a local NGO said:

**I wish that we would be told in advance what is happening. I think because people were not told what to use them for (*Garbage collecting tanks*). People are dumping faeces.**

A 32-year-old male salaried worker added:

**Most of these things…you will just get surprised when they happen. No one is telling us what is going on. Sometimes some call people together like how we are here. We discuss how the community can move forward, but we never hear of that again. We never see them.**

A 60-year-old businessman was more blunt:

**We cannot accept! These people have used us as rubber stamps. They have used the community as rubber stamps. They come, collect information and go. Where do they take it? Maybe it's just someone doing a PhD and is not coming to help the community.**

With regards to project implementation, respondents indicated that CHWs were involved in project activities. A 27-year-old female CHW said:

**I think those who participate mostly the community health worker they participate through the (*chief's*)… office. Because it is the CHWs that do most of the things like health talk, Afya Two (a local NGO)... You find that they involve the CHWs. They inform them.**

A 23-year-old male added that:

**SECODE – an NGO – came up with a group of people, the CHWs. They carried out awareness as far as from Dunga unit to Western unit and all in the line of sanitation and waste management.**

However, some were of the view that engagement with CHWs was only a recent development. A 22-year-old businesswoman said:

**To my understanding, they just started recently; there is an organisation called Concern and Great Lakes. Recently, they just started working with the community health workers. At least they go into the community, and some have talked to the women about so many issues that affect the women.**

### Structures in Community Participation

Participants identified structures in the community that can be used as avenues of engaging the community for better involvement by various stakeholders interested in improving water and sanitation. It was reported that there were organised groups incorporating different criteria: gender, age-group and interests. In terms of gender, the female groups received most mention with participants reporting that there were very few groups consisting of men only. Adult women groups were most popular among the participants, followed by youth groups. Various categories, such as farmers groups, merry-go-rounds, HIV support groups were also mentioned.

With regards to the Youth group, a 23-year-old CHW said:

**So they used their time, they used to take their time, go to a certain place where there is a lot of litter. They collect the litter and burn it.**

A 41-year-old farmer from a women group said:

***Muungano wa Wanakijiji* [Integration of Villagers], which is a community-based organisation, they have participated in… mass awareness, extending the water lines, and giving water guards.**

### Issues in Gender and Disability

Respondents identified gender and disability as playing a major role in participation. The majority of participants believed that more women than men were involved in water and sanitation activities. This was attributed to issues, such as availability and the gender roles in the community, which required the woman to be more involved in water and sanitation activities. A 27-year-old CHW said:

**In most cases, women do take their time and get involved in these interventions.**

This was supported by sentiments from a 20-year-old female, member of a youth group who added that:

**Mostly, the interventions are being conducted by the females in the community more than [their] male counterparts. Women are always available.**

It emerged that gender roles in the community were clearly defined, but also that women were more concerned than men about health matters. A 29-year-old business-woman said:

**…women are so much interested they look after children and look after the father and the whole family.**

A 58-year-old salaried worker said:

**I think females are the best, because females are carers; you see, like men, we have no time to – maybe – to treat our water, so I think it is ladies… in general, because they are more concerned on health than men.**

With regards to people with disabilities, leaders of CBOs admitted to having no member of their group with a disability. A 38-year-old woman, leader of a CBO, said:

**I can say for sure; I don't have anyone who is disabled…But you know they can't move much. It really depends with their level of disability.**

A 20-year-old woman said:

**I think they don't [participate] because they tend to be ignored, they would like to, but since they cannot there is no way.**

### Challenges in Community Participation

Respondents' comments also highlighted the fact that negative attitude from community members can pose as a major setback in effective community participation. This they said can be attributed to a breakdown of information flow from the project managers. This is illustrated by a 43-year-old male farmer who stated:

**I think they are using our people here as rubber stamps to make money and then they go... They get whatever they want, and they disappear.**

A 47-year-old businesswoman added:

**Just recently, we were doing household administration, we went to a house. That house – we were chased away. That's a problem in the community. They think we are going there to watch to observe the house and come back and steal.**

The second aspect mentioned that hinders community participation was poor monitoring and evaluation strategies, which led to project unsustainability. A 30-year-old male salaried worker said:

**The most important is project sustainability. You have planned how to do it. When the project has been put down, it can't pick up… like the government has got a lot of 9 employees for projects… but why they don't follow up?**

A 41-year-old fisherman said:

**NGOs are [partly] to blame to some [extent] because – a good example is Nyalenda Development Group. They were given even equipments by the programme, but they went underground. Some even dispose [of] those things by selling them. You see?**

The third issue mentioned as a challenge to community participation was limited disclosure of study details. This was aptly captured by a 47-year-old business owner who said:

**In some of the implementations which failed, sometimes there was no transparency among the involved people who were to do it … In those which succeeded, those people were transparent, working in teamwork.**

The last aspect mentioned that hinders community participation was overdependence from the community. A 44-year-old woman working as a professional counsellor said:

**I think it is lack of initiative. We don't involve ourselves in these activities; we are waiting for somebody else to bring in the resources may be to help the community… bridge the gap, and after the resource, we don't take the initiative ourselves. We depend so much to be pushed – is when we do these activities. So even though we know that this thing is affecting us we can't do it on our own, we are waiting for the resources to be brought in is when we can do the activities.**

## DISCUSSION

This paper provides qualitative insights into awareness on schistosomiasis and other water and sanitation-related diseases, the water and sanitation situation and issues that affect community participation in water and sanitation as part of community-directed interventions targeting schistosomiasis control in an informal settlement in Kisumu City. It is widely appreciated that the real value of participation emanates from a bottom-up approach where every community member is involved rather than complete non-involvement or individual consultations.^22^

As is often the case with other neglected tropical diseases, awareness of schistosomiasis was low in this setting. Participants indicated that the water and sanitation situation in this setting was generally poor and recommended the utility of several strategies in engaging the community towards improving the water and sanitation situation. The low level of awareness of schistosomiasis in this urban setting was quite unexpected, however, and is in contrast to other previous studies in other bilharzia-endemic regions. For instance, Ndamba and others reported that 80% of villagers in Zimbabwe were aware of schistosomiasis,^23^ whereas other studies in Brazil^24^ and Egypt^25^ revealed that people were fairly familiar with schistosomiasis.

It is likely that the rural versus urban dichotomy might explain the differences observed, especially since schistosomiasis is largely considered a rural phenomenon. Considering the proximity of Nyalenda B to Lake Victoria, it is also plausible that most of the infections observed in this setting are due to active transmission in situ as opposed to the infection being attributable to migrants moving in from the rural areas in search of employment opportunities. Two arguments might support this position: First, if the bilharzia-rural phenomenon was true, migrants from rural areas would be expected to bring with them their awareness (if any). Second, recent research supports active transmission in this setting.^10^ Another explanation for the low awareness may be related to the fact that schistosomiasis is still not considered a significant public health problem compared to other infections, as illustrated by a 23-year-old woman volunteering in a local NGO:

**You see, bilharzia is 1 of the few diseases that are not talked about much. They are compared to things like HIV/AIDS, malaria and all that, ……I think the best thing is that when the community can be sensitised on the fact that the bilharzia is also a problem.**

The subtle morbidity or not easily detectable sequel associated with schistosomiasis may give the wrong impression of a less significant health threat that does not warrant seeking medical attention,^26^ further confounding its level of awareness. On the other hand, it was noteworthy that participants had a fair amoung of knowledge about other waterborne and sanitation-related conditions, such as cholera, amoebiasis, typhoid, and diarrhoea. Like many other informal settlements and as aptly captured by the respondents, Nyalenda B grapples with water and sanitation problems, among them poor drainage systems, low latrine coverage, broken pipes and leakage of the sewerage system. This area has an acute water shortage problem, with only 40% of the population in Kisumu City having access to piped water.^17^ Consequently, a majority of slum dwellers rely on water sources that studies prove to have high degrees of contamination such as springs, the lake and unprotected wells.^27^ Ad ditionally, poor drainage systems and low latrine coverage are attributable to high water tables coupled with black cotton soils and rock outcrops.

Studies show that about 11% of the population living in the slums have no latrines and rely on their neighbours' toilets, the wrap and throw (“flying toilets”) or open defecation in the fields,^21^ leading to high levels of environmental faecal contamination. Further support of this contamination comes from a study that reported *Escherichia coli* contamination in 100% of a subsample of fish (*Rastrineobola argentea*) sold in 6 markets within the City.^28^ As a result, morbidities related to water and sanitation are on the rise such as diarrhoea and intestinal helminthiasis in this setting.^9,29^

A concerted effort is needed by the Municipal council, stakeholders and community members to improve the sewerage infrastructure and improve water treatment and increase access to adequate and safe drinking piped water. A key element of community engagement is participation by the individuals, community-based organisations, and institutions that will be affected by the effort. From the present study, participants identified structures in the community that could be used as avenues of engaging the community in improving water and sanitation situation. Among those that received mentions were: use of organised groups, such as youth groups, gender-based groups, adult women groups, farmers groups, merry-go-rounds, and HIV support groups. Indeed, such partnerships and groups can help mobilise resources and influence systems and serve as catalysts for changing policies, programmes, and practices.^30^ For instance, community- and faith-based organisations (CBOs/FBOs) are a major source of support for millions of families affected by AIDS.^31^

Respondents' comments also highlighted the fact that community participation may take a gender-biased dimension, with women expected to take care of water and sanitation and health-related activities. This gender-biased dimension may stem from the fact that women, in sub-Saharan countries are traditionally expected to take the lead in food production as well as the general health and well-being of the family. Therefore, women are naturally at the heart of home-based care management.^32,33^ However, since water and sanitation or health problems have no gender discrimination, the emphasis should be on the pursuit of gender equity in participation and opportunity.

Ultimately, the consideration of gender issues benefits everyone. Absence or low level of participation from people with disabilities, as seen in our study, may emanate from societal discrimination. Partiality and social intolerance in the community towards people with disability can prove an uphill task for them when they seek to contribute to policy processes or other participatory processes.^34^ Furthermore, stigma can negatively influence their confidence in advocating for their agenda during programme activities or policy-making processes, therefore necessitating special attention where possible.

Management of water and sanitation services may provide valuable opportunities for the employment of youth in infrastructure maintenance programmes. Perhaps 1 of the ways to assess the community's performance on sanitation would involve use of strategies such as community-led total sanitation approach (CLTS) and assessing its impact on re-infection patterns of soil-transmitted helminthiasis, schistosomiasis and other waterborne and sanitation-related diseases. Community participation is a multilevel spectrum that needs to be nurtured. Several factors were mentioned that hinder community participation including negative attitude from community members, poor monitoring and evaluation strategies, which has led to project unsustainability, limited disclosure of project details to community members, and overdependence from the community. Project failures may occur when community members are unable to influence public action through accountability. Lack of transparency often fosters mistrust and misunderstanding between project authorities and local communities, whereas sustainability is often supported by the continuity of leadership.^35,36^

### Limitations

This study employed qualitative methods for data collection and analysis. This means that the participant's responses were the main basis for results and conclusions. Participant's responses may be biased, based on individual perceptions and attitudes towards the various topics of discussion. To control for this, the moderator took a neutral stance to avoid influencing the responses from the participants. Probing technique was also used to capture the participants' full understanding of the topic of discussion.

## CONCLUSION

Our results show that there was low awareness of schistosomiasis in this setting, despite the high prevalence as discovered in other studies,^9^ suggesting that the disease is perceived to be of low public health importance. Poor drainage systems, low latrine coverage, broken pipes and leakage of the sewerage systems were mentioned as the leading factors associated with poor water and sanitation conditions in Nyalenda B. Use of organisational groups and partnerships was cited as an important avenue of engaging community members towards improving water and sanitation activities. Our findings suggest that promotion of and organisational development and sensitisation among community groups could well be effective for improving community involvement in water and sanitation projects. Community participation could be enhanced by attitude changes, information sharing, and consultation to improve monitoring and evaluation as well as transparency and accountability.
